# Age at menarche does not correlate with the endometriosis phenotype

**DOI:** 10.1371/journal.pone.0219497

**Published:** 2019-07-23

**Authors:** Louis Marcellin, Pietro Santulli, Serena Pinzauti, Mathilde Bourdon, Marie Charlotte Lamau, Bruno Borghese, Felice Petraglia, Charles Chapron

**Affiliations:** 1 Université Paris Descartes, Sorbonne Paris Cité, Faculté de Médecine, Assistance Publique – Hôpitaux de Paris (AP-HP), Service de Chirurgie Gynécologie Obstétrique II et Médecine de la Reproduction, Hôpital Universitaire Paris Centre (HUPC), Centre Hospitalier Universitaire (CHU) Cochin, Paris, France; 2 Equipe Génomique, Epigénétique et Physiopathologie de la Reproduction, Département Développement, Reproduction, Cancer, Inserm U1016, Université Paris Descartes, Sorbonne Paris Cité, Faculté de Médecine, AP-HP, HUPC, CHU Cochin, Paris, France; 3 Equipe Stress Oxydant, Prolifération Cellulaire et Inflammation, Département Développement, Reproduction, Cancer, Inserm U1016, Université Paris Descartes, Sorbonne Paris Cité, Faculté de Médecine, AP-HP, HUPC, CHU Cochin, Paris, France; 4 Department of Experimental, Clinical and Biomedical Sciences, University of Florence, Obstetrics and Gynecology, Careggi University Hospital, Florence, Italy; Medway NHS Foundation Trust, UNITED KINGDOM

## Abstract

**Objective:**

To evaluate the association between the endometriosis phenotype and the age at menarche.

**Design:**

An observational, cross-sectional study using prospectively collected data (Canadian Task Force classification II-2).

**Setting:**

Single university tertiary referral center.

**Patients:**

To be eligible, women had to have undergone their 1^st^ complete surgical exeresis of endometriotic lesions. For each patient, a standardized questionnaire was completed the month before the surgery. Endometriotic lesions were classified into 3 phenotypes: superficial peritoneal endometriosis (SUP), endometrioma (OMA), or deep infiltrating endometriosis (DIE). Patients were divided into 3 groups: early menarche (< 12 years), typical menarche (≥ 12 and ≤ 13 years) and late menarche (> 13 years). The groups were compared in terms of general characteristics, medical history, disease phenotype, and disease severity.

**Interventions:**

Surgical management for a benign gynecologic condition.

**Main Outcome Measure(s):**

Correlation between the endometriosis phenotype and the age at menarche.

**Measurements and main results:**

From January 2004 to December 2016, 789 women with histologically confirmed endometriosis were enrolled in the study. The mean age at menarche was 12.9 ± 1.6 years of age, (range 9 to 18). The mean age at menarche and the mean time interval between menarche and the 1^st^ surgery for endometriosis were not significantly different between the three phenotypes (SUP, OMA, DIE). When women with early menarche, typical menarche, or late menarche were compared, no differences were observed in terms of the endometriosis phenotype and the anatomical distribution of the endometriotic lesions.

**Conclusion:**

For women operated for the first time for endometriosis, age at menarche is not associated with the disease phenotype.

## Introduction

Up to 10–15% of women of reproductive age are affected with endometriosis worldwide [1]. Three well-recognized and seemingly different forms of endometriosis are now consistently used as a standard classification of the phenotype [2]: (i) Superficial (or peritoneal) endometriosis (SUP), (ii) Ovarian endometrioma (OMA), and (iii) Deep infiltrating endometriosis (DIE) [3, 4]. The development of lesions, which is thought to be a consequence of ectopic implantation of refluxed menstrual tissue, is the most well-accepted theory for the main causal process [5, 6].

Endometriosis is a hormone-sensitive pathology, and symptoms vary with the menstrual cycle [7]. For girls, the menarche characterizes the transition from childhood to adolescence as the main marker of the timing of puberty that occurs at the average age of 12.8 years in high-income countries [8, 9]. A woman’s exposure to menstruation is increased in case of early menarche. Consequently, according to the “retrograde menstruation” theory, a young age at menarche, via earlier retrograde menstrual flow exposure, may be responsible for an increased endometrium volume of the pelvic cavity. Therefore, this might participate in enhancing the risk of endometriosis. While early menarche (i.e., occurring at less than 11–12 years of age) has been reported to be associated with an increased risk of endometriosis [10–13], other studies have found no such association [14–18]. Such discrepancies may be explained by the heterogeneity of the disease. Studies on the relationship between the age at menarche and the endometriosis phenotypes are lacking.

The aim of the present study was to explore the age at menarche in endometriosis patients and to investigate whether the age at menarche correlates with the histologically proven endometriosis phenotype in a large cohort of women operated for the first time for endometriosis.

## Materials and methods

From January 2004 to December 2016, we conducted an observational, cross-sectional study using a prospectively managed database including data from non-pregnant women who were less than 42-years-old and who had undergone complete surgical exeresis of symptomatic endometriosis at our institution. The local ethics committee (approval number 05–2006 provided by the “Comité Protection des Personnes of Paris-Cochin”) of our institution approved the study protocol. All of the included patients signed a written informed consent form authorizing that the clinical data may be used for scientific epidemiological studies. All of the women operated for the first time and afflicted with histologically proven endometriotic lesions were included in the study. Patients visually diagnosed with endometriosis, but lacking histological confirmation, were deemed ineligible for this study [19]. Previously reported indications for surgery (possibly more than one for each patient) [20] were the following: (i) chronic pelvic pain, defined as the presence, for at least 6 months, of dysmenorrhea and/or intermenstrual pelvic pain and/or dyspareunia of moderate to severe intensity [21]; (ii) infertility, defined as at least 12 months of unprotected intercourse that did not result in pregnancy [22]; (iii) a pelvic mass (e.g., a benign ovarian cysts). Women with cancer, infectious disease, and/or those who refused to provide their consent for participation in the study were excluded.

For analysis purposes, patients with histologically proven endometriotic lesions were classified into three groups: SUP, OMA, and DIE [23]. DIE could be established following radical surgery (e.g., a bowel resection, partial cystectomy, or ureteral resection) when the muscularis (located in either the bladder, intestine, or intrinsic ureter) was found to be infiltrated by endometriotic tissue [24]. For the other locations (i.e., uterosacral ligaments, the extrinsic ureter, or the vagina) DIE was arbitrarily defined as endometriotic tissue infiltrating more than 5 mm beneath the peritoneum surface [25]. Since the three different types of endometriosis frequently occur in conjunction, patients were arbitrarily classified according to their most severe lesion [19]. By definition, patients were ranked from the least to most severe phenotype as follows: SUP, OMA, and DIE [23]. During the surgical procedure, the extent of endometriosis (i.e., the stages and mean scores: total, adhesions, implants) was assessed according to the standards set by the American Society for Reproductive Medicine (ASRM) classification [26].

For each patient, general data were prospectively recorded during face-to-face interviews conducted by the surgeon in the month preceding the surgery, using a structured previously published questionnaire. All of the data were compiled in an anonymous computerized database developed with a data manager and stored on a secure server [23]. All of the surgeons (CC, LM, PS, and BB) who included women in the study employed the same surgical procedures in accordance with the surgical strategy decided during the weekly multidisciplinary meetings prior to the surgery. The following data were collected: age, birth weight, height, weight, body mass index (BMI), history of endometriosis for first-degree relatives, age at menarche, previous obstetrical history (gravidity, parity, voluntary abortion, miscarriages, or ectopic pregnancy), existence and duration of infertility (primary or secondary), menstrual cycle (as “always regular”, “often regular”, or “never regular”), painful symptoms (i.e., dysmenorrhea, deep dyspareunia, non-cyclic chronic pelvic pain, gastrointestinal symptoms, or lower urinary tract symptoms), a prior history of rectorrhagia and/or hematuria, age at the 1^st^ surgery for endometriosis, prior history of uterine surgery (i.e. myomectomy or cesarean-section), use of oral contraceptive pills (OCPs), use of an intrauterine device (IUD), smoking habits, and past history of hormonal treatment for endometriosis. Gastrointestinal symptoms were defined as one or more of the following chronic or menstruation-specific symptoms: diarrhea, constipation, rectorrhagia, proctitis, or colic rectal pain [27]. Similarly, lower urinary tract symptoms were defined as one or more of the following chronic or menstruation-specific symptoms: hematuria, recurrent urinary tract infections, pain while urinating, pollakiuria, non-microbial cystitis, or dysuria [28]. Pain intensity was evaluated preoperatively using a 10-cm visual analog scale (VAS) [29]. For the purpose of this study, the intensity of each preoperative painful symptom was denoted as moderate (VAS < 7) or severe (VAS ≥ 7) [30]. The median age at the onset of menarche for French teenagers is currently 12.8 years of age [9]. Thus, as previously reported [31], in this study early menarche was defined as an age at menarche < 12 years of age, normal menarche as an age at menarche ≥ 12 and ≤ 13 years of age, and late menarche as an age at menarche > 13 years.

First, we described the baseline characteristics of the cohort. Then, in order to verify our hypothesis, comparisons of the age at menarche and the time interval between the age at menarche and the first surgery for endometriosis were conducted according to the three endometriosis phenotypes (i.e., SUP, OMA, and DIE). Because endometrioma and deep endometriosis could co-exist in the DIE group, we compared the age at menarche between women with exclusive OMA to DIE women without OMA. We also assessed the age at menarche according to ASRM classification. Finally, the age at menarche, menstrual cycle characteristics, painful symptoms, and the anatomical distribution of the endometriosis lesions were compared among the study groups according to the age at menarche (early menarche ≤ 12 years, normal menarche ≥ 12 and ≤ 13 years, and late menarche > 13 years). To address this question from a pediatric point of view, we also compared the disease phenotype between very early menarche (< 10 years) to very late menarche (> 15 years).

All of the statistical data were compiled in a computerized database (S1 Dataset). Continuous data are presented as the mean and standard deviation. Student’s t-tests were carried out when appropriate. The Student’s t-test or the Mann-Whitney U-test was used for quantitative variables and the Pearson’s Chi-square or Fisher’s exact test for qualitative variables, as appropriate. When more than two groups were compared, we used the Kruskal-Wallis test. When group medians were significantly different by the Kruskal-Wallis test (p < 0.05), pairwise comparisons were performed with Dunn’s multiple comparison test. Potential confounding variables that were independently associated with the disease phenotype identified in the univariate analysis were added in a multiple linear regression model: body mass index, menorrhagia, and non-cyclic chronic pelvic pain age were included in the multiple logistic regression model. Crude odds ratios (cORs) and 95% confidence intervals (CIs) were calculated. A p-value < 0.05 was considered statistically significant. The statistical analyses were performed using STATA software for Macintosh (Stata/IC 11.0 for Mac, StataCorp College Station, TX, USA).

## Results

**Fig 1:**
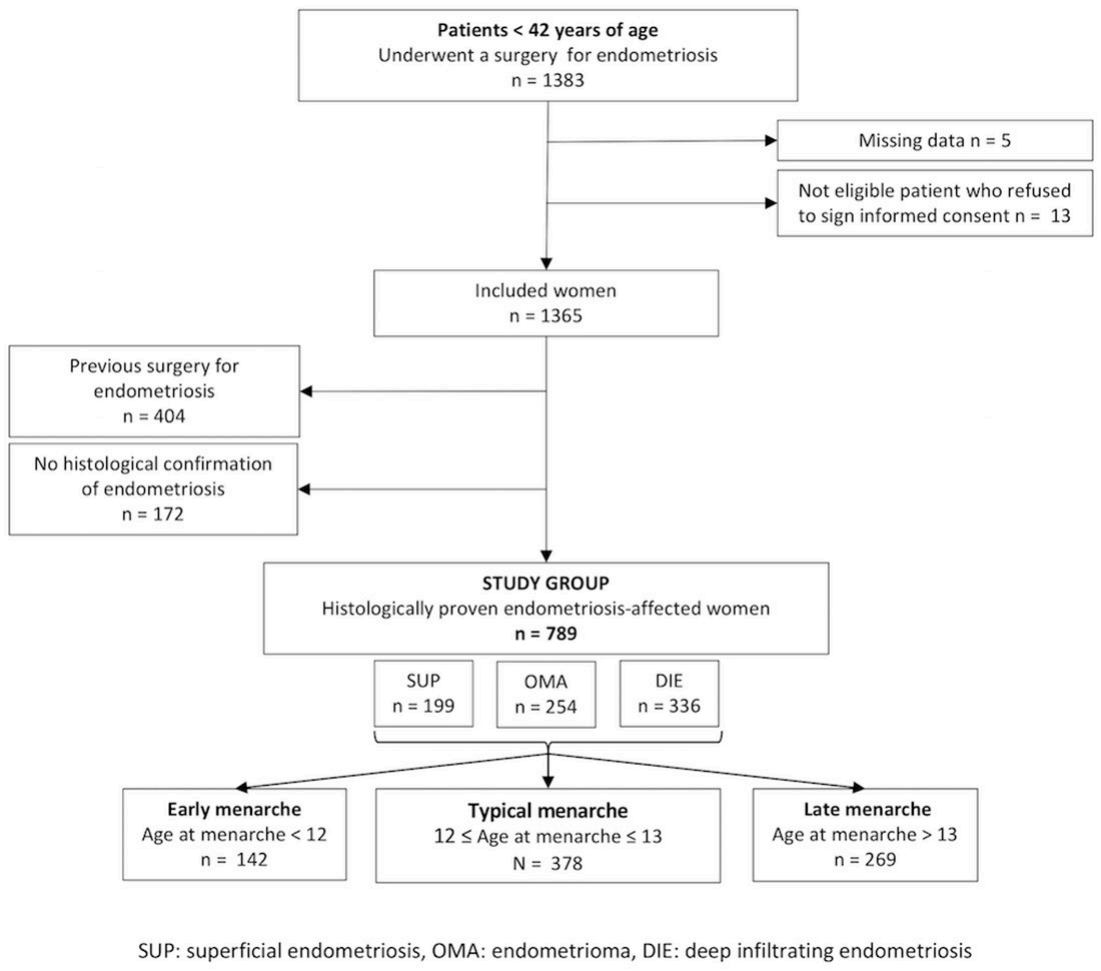
Flowchart outlining the selection process of patients operated for the first time for endometriosis.

The process of our data selection is shown in Fig 1. From January 2004 to December 2016, 1,383 women underwent gynecological surgery for endometriosis at our institution. After exclusion of 5 women with missing data, 13 women who refused to provide their signed informed consent, 404 women with a previous surgery for endometriosis, and 172 women without histological confirmation of endometriosis, 789 women were analyzed. The patient distribution according to the worst endometriotic phenotype was as follows: SUP (199 patients; 25.2%); OMA (254 patients; 32.2%); and DIE (336 patients; 42.6%). According to the age at menarche, the distribution in the early, normal, and late menarche was as follows: early menarche < 12 years (142 patients; 18.0%); typical menarche ≥ 12 and ≤ 13 years (378 patients; 47.9%); and late menarche > 13 years (269 patients; 34.1%).

The mean age of the women of the cohort was 30.9 ± 5.1 years. The mean age at menarche was 12.9 ± 1.6 (range 9–18) years. The rate of nulligravida was 72% (n = 568/789). The patient baseline characteristics are presented in S1 Table.

The mean age at menarche and the mean time interval between menarche and the 1^st^ surgery for endometriosis were not statistically different in SUP, OMA, and DIE group (age at menarche: 13.0 ± 1.7, 12.9 ± 1.5, and 12.9 ±1.5 years, respectively; p = 0.68, and time interval between menarche and the 1^st^ surgery: 17.4 ± 5.6, 18.4 ± 5.5, and 18.0 ± 5.0 years, respectively; p = 0.13).

Among the DIE affected women, no difference was observed for the age at menarche between the women with intestinal DIE lesion and the women with non-intestinal DIE lesions. (12.9 ± 1.6 years [n = 100] vs. 12.9 ± 1.5 years [n = 236], p = 0.90). The age at menarche was not significantly different between the women with endometrioma only and the women with DIE without OMA (12.9 ± 1.5 years [n = 254] vs. 12.9 ± 1.6 years [n = 240], p = 0.66). When the age at menarche was compared according to the ASRM classification, no differences were observed between the four stages (stage I: 12.9 ± 1.6 years [n = 199], II: 13.0 ± 1.7 years [n = 160], III: 12.9 ± 1.5 years[n = 246], and IV:12.9 ± 1.5 years [n = 184], p = 0.16).

Comparison of women’s baseline characteristics between early (< 12 years), typical (≥ 12 and ≤ 13 years), and late (> 13 years) menarche are presented in Table 1. Women with early menarche had a higher BMI (23.0 ± 3.8 vs. 21.6 ± 3.2, and 21.0 ± 3.0, respectively; p < 0.001) and higher rate of menorrhagia (54.2% vs. 47.6% and 42.0, respectively; p = 0.02) than typical menarche and late menarche. No difference was observed regarding the menstrual cycle regularity, school absenteeism, loss of conscientiousness during menses, or OCP use. No difference was observed between the existence of dysmenorrhea, the duration of pelvic pain, and preoperative mean painful symptom VAS scores, except for the non-cyclic chronic pelvic pain score, which was higher in the early menarche group than typical menarche group (3.3 ± 3.3 vs. 2.4 ± 2.9 and 2.5 ± 3.0, p = 0.01) (Table 1). The distribution of the endometriosis phenotype, the presence of endometrioma, the mean number of DIE lesions, and the DIE lesion classification were not different between early (< 12 years), typical (≥ 12 and ≤ 13 years), and late (> 13 years) menarche groups. The rate of severe ASRM stage (III or IV) did not correlate with the early and late menarche age. Early menarche (< 12 years) and late menarche (> 13 years) were not associated with the endometriosis phenotype (Table 2). In a logistic regression multivariable model, after adjustment for the body mass index, menorrhagia, and non-cyclic chronic pelvic pain, early menarche (< 12 years) and late menarche (> 13 years) were still not associated with the endometriosis phenotype (Table 3). No difference was observed in the disease phenotype distribution when very early menarche (n = 51) were compared to very late menarche (n = 149) (SUP: 21.5% (n = 11) vs. 22.5% (n = 33), OMA: 27.5% (n = 14 vs. 24.1% (n = 36), and DIE: 51.0% (n = 26) vs. 53.7% (n = 80); p = 0.90).

**Table 1:** Comparison of the menstrual cycle and the painful symptoms of 789 patients at first surgery for endometriosis, according to early, typical, and late menarche.

	Early menarche (Age at menarche < 12) (n = 142)	Typical Menarche (12 ≥ Age at menarche ≤ 13) (n = 378)	Late menarche (Age at menarche > 13) (n = 269)	*p*
Age y *	30.7 ± 5.6	30.8 ± 5.1	31.1 ± 4.9	0.71
BMI kg/m^2^ *	23.0 ± 3.8 ^b^^,^^c^	21.6 ± 3.2	21.0 ± 3.0	< 0.01
Never used tobacco n (%)	76 (53.5)	162 (42.8)	149 (55.4)	< 0.01
Familial history of endometriosis n (%)	13 (9.2)	34 (9.0)	25 (9.3)	0.99
Nulligravida n (%)	94 (66.2)	283 (74.9)	191 (71.0)	0.08
Regular menstrual cycle n (%)				
• *Always regular*	113 (79.6)	314 (83.1)	211 (78.4)	
• *Often regular*	1 (0.7)	11 (2.9)	5 (1.9)	
• *Never regular*	28 (19.7)	53 (14.0)	53 (19.7)	
Menorrhagia n (%)	77 (54.2)	180 (47.6)	113 (42.0)	0.02
OCs treatment n (%) ^a^				
• *Never*	19 (12.3)	48 (12.7)	33 (12.3)	
• *Current user*	81 (57.1)	227 (60.1)	165 (61.4)	
• *Previous user*	42 (29.6)	103 (27.2)	71 (26.4)	0.95
Age of first OCP prescription y *	17.9 ± 3.6	18.4 ± 3.4	18.9 ± 3.3	0.07
History of school absenteeism n (%)	47 (33.1)	111(29.4)	73 (27.1)	0.45
History of loss of consciousness during menses n (%)	16 (11.3)	45 (11.9)	34 (12.6)	0.91
Duration of pelvic pain m *	41.0 ± 48.1	37.7 ± 43.9	45.5 ± 54.8	0.97
Dysmenorrhea n (%)				
• *No dysmenorrhea*	18 (12.7)	49 (13.0)	41 (15.3)	
• *Primary*	77 (54.2)	200 (52.9)	137 (50.9)	
• *Secondary*	47 (33.1)	129 (34.1)	91 (33.8)	0.91
Painful symptoms mean VAS scores *				
• *Dysmenorrhea*	6.8 ± 2.8	6.5 ± 2.6	6.4 ± 2.7	0.38
• *Deep dyspareunia*	3.6 ± 3.4	3.7 ± 3.3	3.8 ± 3.4	0.86
• *Non-cyclic chronic pelvic pain*	3.3 ± 3.3 ^b^^,^^c^	2.4 ± 2.9	2.5 ± 3.0	0.01
• *Gastrointestinal symptoms*	3.1 ± 3.5	3.1 ± 3.5	2.8 ± 3.4	0.55
• *Lower urinary tract symptoms*	1.0 ± 2.4	1.1 ± 2.5	0.8 ± 2.1	0.24
Infertility n (%)				
• *Primary*	29 (20.4)	91 (24.1)	67 (24.9)	
• *Secondary*	15 (10.5)	26 (6.9)	21 (7.8)	0.64
Duration of infertility m *	40.9 ± 39.4^b^	29.4 ± 18.4	30.8 ± 22.6	0.03

* Mean ± SD

^a^ less than 2% of missing data, BMI: body mass index, OCP: oral contraceptive pill, VAS: visual analogic scale, y: year, m: month,

^b^ significantly different when compared to typical menarche (p < 0.05)

^c^ significantly different when compared to late menarche (p < 0.05)

**Table 2:** Comparison of the disease phenotypes and the anatomical distribution of the lesions of 789 patients at first surgery for endometriosis, according to early, typical, or late menarche.

	Early menarche (Age at menarche < 12) (n = 142)	Typical Menarche (12 ≥ Age at menarche ≤ 13) (n = 378)	Late menarche (Age at menarche > 13) (n = 269)	*p*
**Endometriosis phenotype** n (%)				
• SUP	37 (26.0)	88 (23.3)	74 (27.5)	
• OMA	43 (30.3)	124 (32.8)	87 (32.3)	
• DIE	62 (43.7)	166 (43.9)	108 (40.2)	0.75
**Endometrioma** n (%)				
• Presence	63 (44.4)	174 (46.0)	117 (43.5)	0.81
○ Bilateral	10 (7.0)	38 (10.5)	36 (13.4)	
○ Right	19 (13.4)	50 (13.2)	31 (11.5)	
○ Left	34 (23.9)	86 (22.8)	50 (18.6)	0.41
**Deep infiltrative endometriosis**				
• Mean number of DIE *	2.1 ± 1.5	2.3 ±1.7	2.5 ± 1.7	0.41
• Number of DIE lesion				
○ *n = 1*	30 (21.1)	68 (17.9)	43 (16.0)	
○ *n = 2*	12 (8.4)	49 (13.0)	26 (9.7)	
○ *n ≥ 3*	20 (14.1)	49 (13.0)	39 (14.5)	0.59
**Patient surgical classification n (%)** *According to the surgical classification for DIE [42]*				
• USL	26 (41.9)	63 (37.5)	39 (35.2)	
• Vagina	6 (9.7)	19 (11.5)	17 (15.7)	
• Intestine	18 (29.0)	49 (29.5)	33 (30.5)	
• Bladder	7 (11.3)	21 (12.7)	7 (6.5)	
• Ureter	5 (8.1)	14 (8.4)	12 (11.1)	0.71
**ASRM Stage n (%)**	73 (51.4)	211 (55.8)	143 (53.1)	0.62
• I	39 (27.5)	98 (25.9)	68 (25.3)	
• II	31 (21.8)	70 (18.5)	58 (21.5)	
• III	44 (31.0)	112 (29.6)	86 (31.9)	
• IV	28 (19.7)	98 (25.9)	57 (21.2)	0.69
**ASRM score** * According to the American Society for Reproductive Medicine [26].				
• Mean total ASRM score	25.8 ± 26.8	27.0 ± 26.2	24.8 ± 25.0	0.57
• Mean implant ASRM score	12.9 ± 11.2	13.9 ± 11.7	13.7 ± 12.2	0.68
• Mean adhesion ASRM score	13.3 ± 20.1	13.3 ± 18.8	11.1 ± 17.0	0.26

* Mean ± SD; SUP: superficial endometriosis, OMA: endometrioma, DIE: deep infiltrating endometriosis

**Table 3:** Age at menarche and endometriosis: Comparison according to the disease phenotype (SUP, OMA, and DIE).

	SUP (n = 199)		OMA (n = 254)		DIE (n = 336)		*p*
Age at menarche OR [95%CI]	**OR**	**95% CI**	**OR**	**95% CI**	**OR**	**95% CI**	
• Typical menarche (≥ 12 and ≤ 13 years)	1	Reference	1	Reference	1	Reference	
• Early menarche (< 12 years)	1.2	0.8–1.9	0.9	0.6–1.4	0.9	0.6–1.4	ns
• Late menarche (> 13 years)	1.3	0.9–1.8	0.9	0.6–1.2	0.9	0.6–1.2	ns
Age at menarche aOR* [95%CI]	aOR*	**95% CI**	aOR*	**95% CI**	aOR*	**95% CI**	
• Typical menarche (≥ 12 and ≤ 13 years)	1	Reference	1	Reference	1	Reference	
• Early menarche (< 12 years)	1.2	0.8–2.0	1.0	0.6–1.6	0.8	0.6–1.30	ns
• Late menarche (> 13 years)	1.3	0.9–1.9	0.9	0.6–1.2	0.9	0.6–1.2	ns

SUP: superficial endometriosis, OMA: endometrioma, DIE: deep infiltrating endometriosis, y: years

* Adjusted for body mass index, menorrhagia, and non-cyclic chronic pelvic pain

## Discussion

In this large series, the age at menarche and the time interval between menarche and the 1^st^ surgery for endometriosis did not correlate with the disease phenotype (SUP, OMA, or DIE). No difference was observed in the menstrual cycle characteristics, painful symptoms, or the anatomical distribution of endometriosis between early, typical, and late menarche.

The strength of this study lies in its methodological design. This is the largest study (*n* = 789) to address whether age at menarche correlates with the endometriosis phenotype. This large hospital-based study, in which all of the patients had a surgical diagnosis and histological confirmation, reduced the risk of selection bias. Following surgery, the patients were classified according to their most severe lesion (ranked from SUP, OMA, to DIE), thereby allowing precise analysis of a correlation between age at menarche and the time interval from menarche to the 1^st^ surgery for endometriosis and the disease phenotype. Despite the extensive precautions that we took, our study might have the following shortcomings and/or biases. The study was conducted in a referral center specializing in endometriosis surgery. This may contribute to an elevated rate of patients affected by severe forms of endometriosis. Our study only included women with surgically diagnosed endometriosis. Women with asymptomatic forms of endometriosis [32] were, therefore, not considered in this study. In addition, a potential recall bias is induced by the fact that the women reported their age at menarche a long time after the event. In a prospective cohort of 1050 patients, Cooper et al. studied the level of validity of self-reported age at menarche at adulthood compared to when it is reported at adolescence. They found that only 43.6% of the patients had provided exactly the same age at menarche when they were 48 years of age as had been recorded during their medical examination at 14–15 years of age [33]. However, in our study, the women were younger than 48 years old (mean age at first surgery = 31.6 ± 5.5 years), which may limit the recall bias. However, recollection in women who have suffered from chronic pain symptoms is not as reliable [34] and there is no evidence that recollection is better just because women are younger.

To study the association between age at menarche and the endometriosis phenotype we designed a cross-sectional study including endometriosis-affected women while using the SUP phenotype as a reference. A control group of non-endometriotic women with negative laparoscopy would not be appropriate because, instead of being healthy women, these patients suffered from a sufficient degree of non-endometriosis gynecological pathology to warrant pelvic surgery. In several studies, control women with diminished functional ovarian function [35] and uterine myoma [36–38] have been significantly associated with a younger age at menarche. Conversely, patients with polycystic ovary syndrome are significantly associated with an older age at menarche [39].”

The age at menarche and the time interval between menarche and the 1^st^ surgery for endometriosis are anamnestic items used in daily gynecologic practice. In light of our results, both of these items do not appear to be relevant for determining the disease phenotype of endometriosis. In their meta-analysis (including 18 case-control studies, involving 3,805 cases of surgically diagnosed endometriosis and 9,526 controls) Nnoaham et al. observed a very modest increased risk of endometriosis with early menarche when only studies with good methodological qualities and adequate control of potential confounders were considered. Eleven studies defined early age at menarche as < 12 years old, and in four studies it was defined as ≤ 12 years old. Ten studies were well controlled for confounders. Finally, the authors did not conclude that there was strong evidence for the clinical utility of a history of early menarche in the evaluation of endometriosis [31].

Diagnosing endometriosis is a challenge and tenuous process due to the lack of pathognomonic signs and effective clinical markers [40]. Given the high prevalence of endometriosis in the general population, reliable clinical markers would presumably help clinicians to screen more easily and promptly detect women with painful symptoms at an early stage. For example, anamnestic factors recorded in medical history questioning such as cycle length, duration of flow, and nulliparity have consistently been shown to be possible risk factors for endometriosis [41]. In addition, primary dysmenorrhea and absenteeism from school during menstruation during adolescence have also been identified as markers of DIE [23]. However, the age at menarche in our study did not correlate with the endometriosis phenotype (SUP, OMA, and DIE). New markers are, therefore, needed to suspect an endometriotic phenotype in symptomatic women.

## Conclusion

We did not observe any difference in the age at menarche or the time interval between menarche and the 1st surgery for endometriosis among the different endometriosis phenotypes. In terms of the endometriosis pathogenesis, these epidemiological results indicate a probable lack of phenotype transition. Our results highlight the need to conduct studies: (i) to find clinical markers in the questioning that can help reduce the delay in diagnosing endometriosis; and (ii) to allow for an earlier diagnosis of the endometriosis phenotype.

## Supporting information

S1 Dataset Clinical and anatomical data. (XLSX)

S1 Table Patient baseline characteristics. (DOC)
