# Supplementary material for: Age at menarche does not correlate with the endometriosis phenotype
Source: PLoS One. 2019 Jul 23;14(7):e0219497. doi: 10.1371/journal.pone.0219497 (PMC6650141; doi:10.1371/journal.pone.0219497)
Supplement: S1 Table — (DOC) [file pone.0219497.s002.doc]

**S1 table .**

**Patient baseline characteristics**

|  | **Whole population**  (n = 789) |
| --- | --- |
| Age at menarche y (range) **^*^** | 12.9 ± 1.6 (9-18) |
| Age y **^*^** | 30.9 ± 5.1 |
| BMI kg/m^2^ **^*^** | 21.6 ± 3.3 |
| Never used tobacco n (%) | 439 (55.9) |
| Familial history of endometriosis n (%) | 72 (9.1) |
| Nulligravida n (%) | 568 (72.0) |
| Regular menstrual cycle n (%) |  |
| - *Always regular* | 638 (80.9) |
| - *Often regular* | 17 (2.1) |
| - *Never regular* | 134 (17.0) |
| Menorrhagia n (%) | 370 (46.9) |
| OCPs treatment n (%) |  |
| - *Never* | 100 (12.6) |
| - *Current user* | 473 (60.0) |
| - *Previous user* | 216 (27.4) |
| Age of first OCP prescription y **^*^** | 18.5 ± 3.4 |
| History of school absenteeism n (%) | 231 (29.3) |
| History of loss of consciousness during menses n (%) | 95 (12.4) |
| Length of pelvic pain m **^*^** | 41.0 ± 48.8 |
| Dysmenorrhea n (%) |  |
| - *No dysmenorrhea* | 108 (13.7) |
| - *Primary* | 414 (52.5) |
| - *Secondary* | 267 (33.8) |
| Painful symptoms mean VAS scores ± SD |  |
| - *Dysmenorrhea* | 6.5 ± 2.7 |
| - *Deep dyspareunia* | 3.7 ± 3.5 |
| - *Non-cyclic chronic pelvic pain* | 2.6 ± 3.0 |
| - *Gastrointestinal symptoms* | 3.0 ± 3.4 |
| - *Lower urinary tract symptoms* | 0.9 ± 2.4 |
| Infertility n (%) | 260 (32.4) |
| - *Primary* | 187 (23.7) |
| - *Secondary* | 62 (7.8) |
| Length of infertility m **^*^** | 31.9 ± 25.1 |

^*^  Mean ± SD

BMI: body mass index, OCP: oral contraceptive pill, VAS: visual analogic scale, y: year,
